# Task-Related Suppression of the Brainstem Frequency following Response

**DOI:** 10.1371/journal.pone.0055215

**Published:** 2013-02-18

**Authors:** W. David Hairston, Tomasz R. Letowski, Kaleb McDowell

**Affiliations:** Human Research and Engineering Directorate, United States Army Research Laboratory, Aberdeen Proving Ground, Maryland, United States of America; College of Tropical Agriculture and Human Resources, University of Hawaii, United States of America

## Abstract

Recent evidence has shown top-down modulation of the brainstem frequency following response (FFR), generally in the form of signal enhancement from concurrent stimuli or from switching between attention-demanding task stimuli. However, it is also possible that the opposite may be true – the addition of a task, instead of a resting, passive state may suppress the FFR. Here we examined the influence of a subsequent task, and the relevance of the task modality, on signal clarity within the FFR. Participants performed visual and auditory discrimination tasks in the presence of an irrelevant background sound, as well as a baseline consisting of the same background stimuli in the absence of a task. FFR pitch strength and amplitude of the primary frequency response were assessed within non-task stimulus periods in order to examine influences due solely to general cognitive state, independent of stimulus-driven effects. Results show decreased signal clarity with the addition of a task, especially within the auditory modality. We additionally found consistent relationships between the extent of this suppressive effect and perceptual measures such as response time and proclivity towards one sensory modality. Together these results suggest that the current focus of attention can have a global, top-down effect on the quality of encoding early in the auditory pathway.

## Introduction

The auditory brainstem response (ABR) represents some of the earliest encoding of acoustic information within the auditory system, arising from the pooled synchronized response of brainstem neurons. The frequency following response (FFR) component of the ABR represents a phasic, sustained response generally assumed to arise from phase-locking cells within the rostral brainstem projecting into the auditory midbrain, characterized by a frequency profile matching that of the incoming sound [1]–[7]. As a result, the FFR can be used as a metric for encoding quality of sound as represented early in the auditory system. Because the ABR can be recorded passively using scalp electrodes, it has been advocated as an excellent means for assessing low-level auditory function in a non-invasive manner in either a clinical or laboratory setting (see [1] for a review).

Despite its low-level origins, there is evidence that the FFR is malleable based on higher-level cognitive influence. For instance, simultaneous presentation of matching visual information with the ABR eliciting acoustic stimuli leads to an enhanced response [8]–[10], with correlated responses at the cortical level [10]. Meanwhile, within a single recording session, adaptation in response amplitude can occur based on the local acoustic statistics of the stimuli used [11], similar to other examples of experience-dependent modulation [12]–[16] but on a more rapid scale, further supporting malleability from top-down factors.

These types of effects are most likely linked to the direction of attention, which has been extensively shown to modulate cortical responses [17]. For instance selective attention to the auditory domain leads to increased cortical response amplitude and decreased response latency, occurring even in primary cortices [18]–[20], functionally increasing the gain of the attended signal. Alternatively, recent evidence has suggested enhanced selectivity within auditory cortex by inhibiting responsiveness to non-relevant stimuli or specific features [21]–[24]. Similar attention-related filtering mechanisms have been observed when looking across modalities as well [25]–[27].

Although most attention-related research focuses on cortical-level interactions, there is evidence of attention-related mediation of the FFR in both amplitude and latency [28]–[30]. For example, attending to vowels leads to an amplitude increase of the fundamental frequency [29]. In one particular case [28] it has been argued that using a task to focus attention within the auditory modality provides a stronger response than when the task uses an opposing sense (e.g., vision).

To date, however, it remains unclear whether this level of subcortical auditory encoding can also be *suppressed* based solely on the current focus of attention. That is, top-down interactions may also be able to operate in an inhibitory manner when the scenario necessitates it, as a mechanism for filtering non-relevant information. Several examples of this have been shown cortically, especially when looking across modalities, such as where presentation of a stimulus in one sensory modality (e.g. visual or somatosensory) can lead to a decrease in regional measures of neuronal activity in other cortices (e.g. auditory or visual, respectively) [27], [31]–[34]. This paucity is due in part to the fact that most studies of attention effects on early auditory encoding use the ABR-eliciting stimulus as part of the attention-mediating task, making it difficult to separate attention to the response eliciting stimuli from the task itself. Additionally, a non-task “resting” baseline is needed for assessing potential *decreases* from the additional task load.

In this study, we sought to investigate this possibility through the use of a paradigm more common for studies of cortical processing of non-task relevant information [35]. Specifically, participants perform a task in either the visual or auditory modality, where the stimuli used to assess FFR acuity remain in the background, completely irrelevant to the task at hand. Additionally, we include a non-task “baseline” in order to assess potential changes in FFR clarity related to the addition of the task paradigm. We then examine within- versus cross-modality effects on the suppression of low-level auditory encoding, and follow this with an analysis of some behavioral metrics of sensory bias which help predict the extent to which the FFR signal mediation is observed across subjects.

## Materials and Methods

### Participants

Participants were twenty-three adults (6 females) between 22–45 years of age, with normal or corrected-to-normal vision and normal hearing as determined by 250 to 8,000 Hz pure tone air conduction audiometry (25dB or less hearing level at each frequency). All were native English speakers, and none reported as being “fluently bilingual”. While three participants reported having >3 years of musical experience, none performed professionally or had formal training beyond high school. All methods were approved by the Institutional Review Board and conformed to the 1964 Declaration of Helsinki. The voluntary, fully informed consent of the persons used in this research was obtained as required by U.S. Army human use regulations (U.S. Department of Defense, 1999; U.S. Department of the Army, 1990). Data from 5 participants were removed from the group analysis due to technical failure (1), excessive motion (1), or poor SNR (3).

### Experimental Paradigm

#### Primary Tasks

Three different experimental conditions were used; an auditory task, a visual task, and no-task “baseline” condition, each tested in a separate block lasting approximately 7 minutes. At the beginning of each run, the participant was given instructions denoting which task type would be performed. Each paradigm type (visual, auditory, no-task) was performed twice in an order randomized across subjects, with the only caveat that no task type was repeated before all three conditions had been completed at least once.

Subjects were given sensory tasks with the goal of ensuring that they must focus on one particular sensory modality, while also providing behavioral measures of performance. Both sensory tasks were a temporal discrimination presented in a two-interval, forced choice format, designed to be analogous for both sensory modalities. In the *visual task*, subjects were presented with a light grey square centered on the screen, which appeared and disappeared twice. One presentation always lasted 250 ms, and the other was presented within a range of 255–400 ms duration, with order randomized across trials. In the *auditory task*, the target stimulus was a pure tone (587 Hz), also presented once for 250 ms and once for 255–400 ms. In both cases, participants were given 2 seconds to answer which of the presentations was shorter (first or second). In the *no-task* condition, subjects simply fixated on a constant, central cross, identical to one used for the two active tasks.

To ensure that subjects constantly paid attention across the entire block of trials, the ISI between trials varied randomly within a range of 3000–12000 ms (mean 6750 ms) so they could not predict the onset of the next stimulus. The length of the longer stimulus was set to a range (+/−16.7 ms) centered around each participant's own individually-derived 75% discrimination threshold for the task. Discrimination threshold values were acquired prior to testing using an adaptive staircase procedure [36] adapted for the E-Prime programming environment [37]. Using the adaptive staircase and individual-specific concomitant values both provided an assessment of sensory temporal acuity for each subject and modality, and ensured approximately equal difficulty across subjects and conditions. Background tones (see below) were also included during the staircase procedure so as to match the primary testing environment.

#### Background Tones

**Figure 1:**
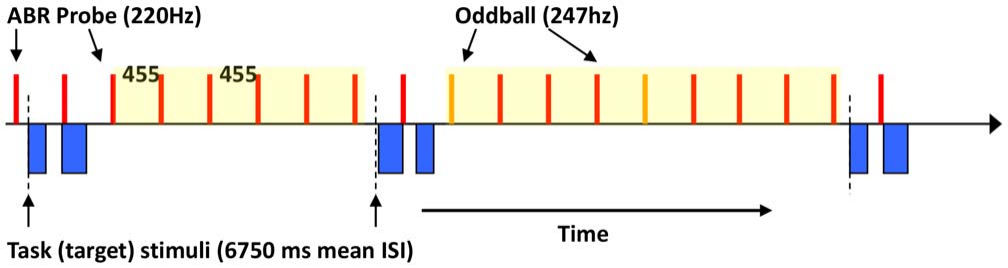
The experimental paradigm. Non-task relevant ABR-eliciting stimuli per presented every 455 ms, with task target stimuli occurring asynchronously, randomly presented (6750 ms mean ISI). Only background tones falling between task stimuli (shaded area) were used in the analysis.

During all conditions, there was an additional background, ongoing “probe” stimulus (1,200 repetitions) used for eliciting the frequency following response (FFR) component of the auditory brainstem response (ABR). The probe was a 100 ms, 220 Hz pure tone (5 ms linear amplitude ramps at on/offset) presented every 455 ms. Occasionally (3% of presentations) the tone changed in pitch (247 Hz); response to this oddball is not discussed here. Participants were told not to worry about the background tones, and focus only on the primary task. By using this interleaved paradigm of both primary and background tasks, we are able to infer responses to both task relevant and task non-relevant stimuli. Figure 1 shows an example of the stimulus paradigm. Background tones occurred with a constant ISI in order to minimize distraction from the primary task.

All sounds were presented via Etymotic Research ER-3A insert tube earphones, presented at 83dB (peak) at the ear. Visual stimuli were presented on a Dell 24” 2408WFP monitor, with central fixation approximately 50 cm from the participant's nose. Data were collected in an acoustically isolated chamber with reflective attenuation. During all 6 testing runs, whole-scalp EEG data were collected using a 64 channel Biosemi active electrode biopotential measurement system, sampled at 8.192 kHz, filtered at 0.1–4 kHz with external references set to bilateral earlobes.

### Analysis

#### Brainstem FFR

Because the brainstem FFR is strongest at scalp location CZ when using bilateral stimuli and linked earlobe reference [1], that served as the primary focus for this initial study. To derive the ABR FFR, EEG data were filtered between 80 and 2,000 Hz using a Butterworth filter, and epoched based on the consistent background tone for the period −200 to 225 ms relative to stimulus onset. Artifacts were rejected based on a +/−35 µV voltage threshold (typically ∼3%), and data from similar blocks (e.g. 2 auditory task runs) were combined. Any cases overlapping the primary task stimuli (∼250) were also removed, and responses to the oddball background tone (247 Hz) were ignored, ensuring all waveforms represented only the auditory response to the consistent non-relevant probe. For *no-task* data, we matched the number and temporal placement of ABR-eliciting events by removing the same cases which overlapped with task stimuli in the other two conditions. Waveforms (typically about 1800 trials remaining) were averaged to yield a phasic FFR for each subject and task type (*visual, auditory, no-task*).

Waveform averages were imported into the Brainstem Toolbox from Northwestern University [1] and used to compute two primary metrics. First, *pitch strength* was calculated as the mean of results from a running autocorrelelogram (40 ms window, 1 ms steps) across the time-locked average EEG waveform during the central 75 ms of response [38], [39]. Thus, it summarizes how consistent the periodicity of the neural response is (but without specificity to a certain frequency), in this case reflecting the highly phasic characteristics of the external acoustic waveform. Note that this is a characterization only of the encoding characteristics, and it distinct from higher cortical processes such as pitch perception which might depend on changes in temporal envelope or more complex features. Second, *frequency response amplitude* was determined by the amplitude of the average response (normalized to nV) of the Fourier-transformed signal within the target frequency range (210–230 Hz), thus reflecting the overall power contained within the target frequency of the phasic response but irrespective of minor temporal fluctuations in that period. Response amplitudes were normalized to the pre-stimulus period to account for small but non-significant differences in baseline. Data from three subjects for whom the SNR (calculated as the level of measured response divided by the pre-stimulus period amplitude) during the *no-task* condition was below 1.5 were removed prior to any analyses.

A slight harmonic distortion was created by the sound presentation equipment (ER3A transducer and tube inserts), as measured by a KEMAR mannequin (GRAS Sound & Vibration) inside the ear when compared to direct recording from the PC sound card. While the FFR response to this harmonic effect is evident in group spectrograms, it is outside of the frequency realm focused on for analyses here.

#### Behavioral Responses

Two primary behavioral metrics were measured. First, for each sensory modality (vision and audition), we used the resulting mean SOA between primary task stimuli from the staircase threshold procedure as an assessment of temporal processing acuity for that sense. Note that in this case, lower values denote a higher acuity – less time necessary to perform the task at equivalent accuracy. From these, a “sensory bias” score was calculated as the relative difference (in ms) when the auditory threshold was subtracted from the visual threshold. This yields positive values indicating higher acuity in audition than vision, while negative scores denote subjects with an acuity bias towards the visual domain.

Second, reaction time (RT) was calculated as the median time, in ms, to press the button relative to the onset of the second of the two primary task stimuli. RTs from the auditory task were subtracted from visual, so that positive values denote faster auditory responses, while negative values (in ms) represent faster responses with a visual, rather than auditory, task. This RT difference (in ms) represents the performance gain by performing the primary task in an opposite modality (visual task, auditory background) versus the same modality (auditory task, auditory background) as the non-relevant stimuli. Additionally, it normalizes RTs across subjects to account for the large variability in general RT tendencies and places all subjects within a common scale.

## Results

### Brainstem Response

**Figure 2:**
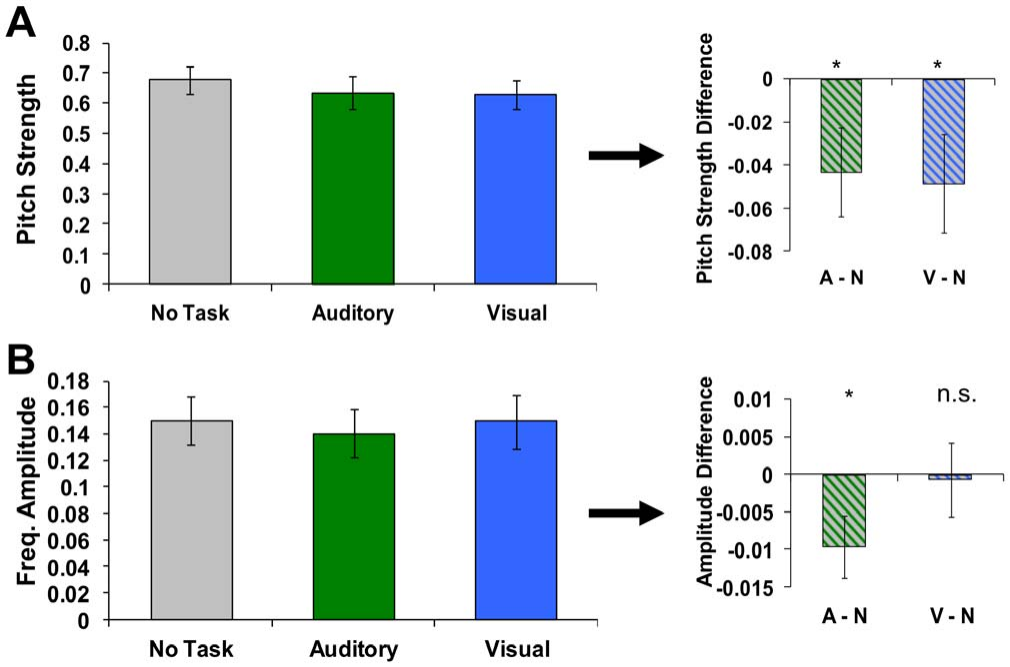
FFR metrics for each task type. Pitch strength significantly decreased for both *visual* and *auditory* tasks (**A**) relative to baseline (no task). In addition, average signal amplitude within the target frequency (220 Hz) decreased for auditory, but not visual tasks (**B**). Error bars represent standard error across subjects Asterisks on right denote significant (p<.05) differences. Note that only background stimuli not overlapping with the task and identical in all conditions were used in this analysis, suggesting a tonic suppression of background auditory signals that may be greater when also performing an auditory task.

For the group of subjects, pitch strength, a measure of the consistency of frequency encoding, was significantly decreased for both *visual* and *auditory* tasks (Figure 2, **A**) relative to a resting (no task) baseline [*visual,* t(16) = 2.12, p<.05, *auditory,* t(16) = 2.10, p<.05)]. In other words, with the addition of the task, brainstem encoding of background, non-relevant signals was less consistent (relative to the actual acoustic signal) than when there was no task. Meanwhile, average signal amplitude within the target frequency (220 Hz) decreased for auditory, but not visual tasks [Figure 2, **B**, *visual,* t(16) = 0.14, p>.05, *auditory,* t(16) = 2.31, p<.05], suggesting a suppression of background auditory signals that is more specific to also performing an auditory (as opposed to visual) task within this domain.

**Figure 3:**
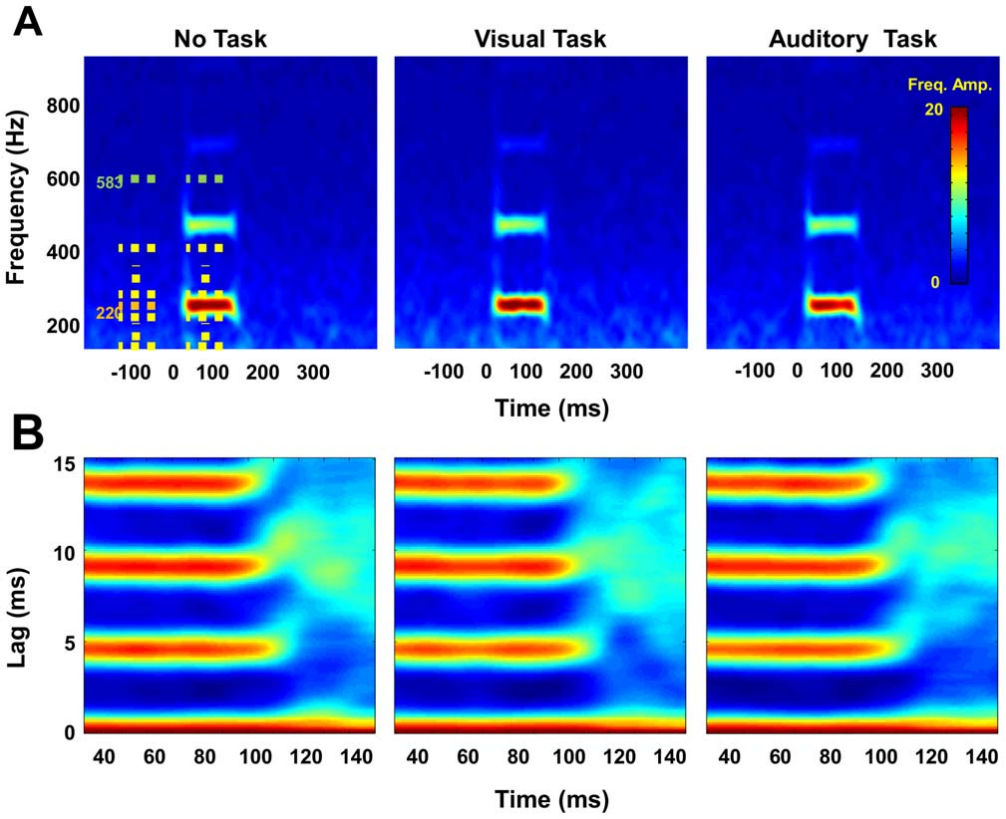
Fourier time-frequency plots for each of the primary conditions. The strongest response occurs primarily within the 220 Hz frequency range, although a short broadboand response is seen at onset and offset in the response spectrogram (**A**). Differences across conditions were limited to only the primary frequency. Dashed lines represent time and frequency periods used for ANOVA; 100–200 Hz (yellow), 210–230 Hz (target range, orange), 240–400 Hz (yellow), and 553–613 Hz (green), pre- and post-stimulus onset. (**B**) shows group average autocorrelelograms reflecting the highly consistent response with this stimulus.

Importantly, this suppressive effect appears within only the primary frequency range of the background FFR-eliciting tone. Figure 3 shows Fourier time-frequency plots (using a 40 ms sliding window with 1 ms steps across the range from 100–900 Hz in 2 Hz increments) for each of the primary conditions. Note there is a substantial response within the 220 Hz range, with transient broadband responses around the beginning and end of each response, but differences across conditions are limited only to the primary frequency tested here.

Statistically, we tested the specificity of this effect by computing the Fourier power within several frequency ranges for a 50 ms window, both pre- and post stimulus and illustrated by dashed lines in Figure 3. These included 100–200 Hz (yellow), 210–230 Hz (target range, orange), 240–400 Hz (yellow), and 553–613 Hz (green). This latter range centers around the frequency of the auditory task stimuli (587 Hz). Strength of the response was calculated as the difference between the pre- and post-stimuli 50 ms windows. Despite slightly different pre-stimulus baselines, in all three tasks ANOVAs show a significant response across the entire frequency spectrum, including below the target range (100–200 Hz, F(16,1) = 27.8, p<.001), above (240–400 Hz, F(16,1) = 17.6, p<.001), and the specific frequency of the auditory task stimuli (583 Hz, F(16,1) = 18.9, p<.001).

However, only the target frequency (220 Hz) showed a significant interaction (F(15,2) = 3.72, p<.05) with task modality for the response period, where the amplitude of the response during the auditory task was lower than in the other conditions. None of the non-target frequency sidebands (100–200 Hz, 240–400 Hz, or 553–613 Hz) showed this effect, highlighting the specificity of the effect to the range of the background tones and not as a broad suppression of all acoustic frequencies.

### Behavioral Responses

**Figure 4:**
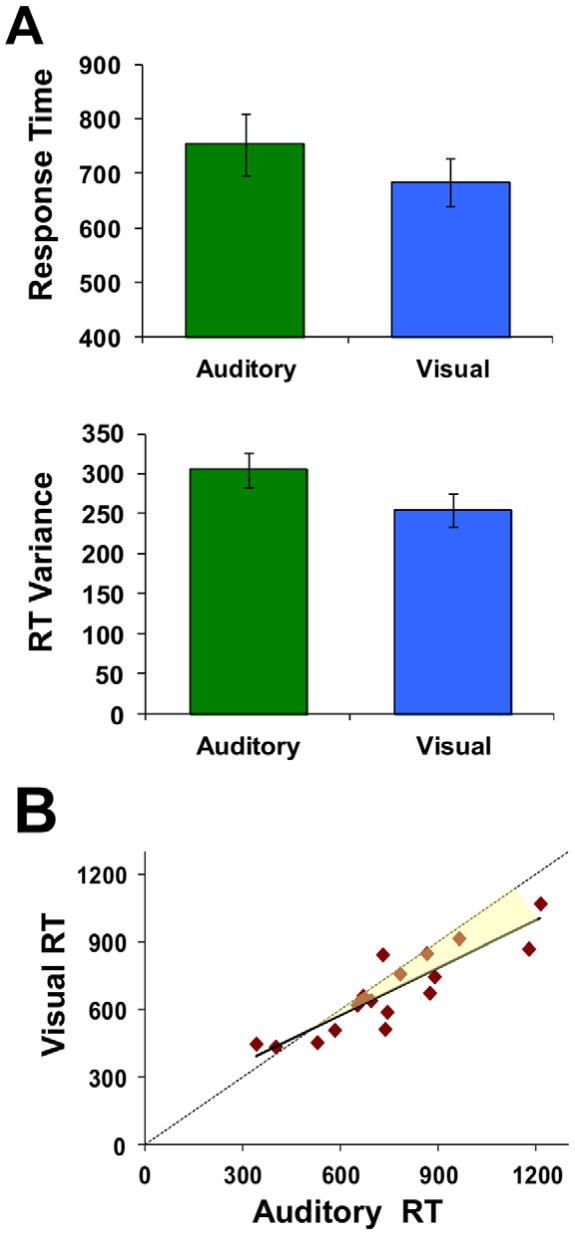
Response time per sensory modality. Subjects responded faster (lower median response times) and more consistently (lower mean within-subject variance) with visual than auditory task stimuli (**A**). Error bars depict standard error across subjects. There was a high correlation across subjects' response times between the two tasks (r = 0.883, p<.05) such that subjects with longer responses during the visual task also tended to take longer to respond with auditory targets as well (**B**). The shaded area emphases how this relative difference increases consistently across subjects.

Because of cross-subject variance observed in the ABR suppression effect described above (e.g. average standard error size covers over 12% of the response amplitude), we sought to examine behavioral metrics which might similarly vary across individuals and help explain this variance. No significant differences were observed in subjects' overall accuracy between the tasks (77.7%+/−2% *auditory*, 80.2%+/−2% *visual*), suggesting an equal, moderate level of difficulty for both tasks, and consistent with having thresholds matched according to the staircase procedure. Subjects did respond faster (684+/−44 ms vs 755+/−56 ms, t(16) = 2.67,p<.05), Figure 4, **A**) and more consistently (lower mean within-subject variance) (254.5+/−20.3 vs 305.0+/−22, t(16) = 5.84,p<.05) when performing the visual task with auditory background, than when the task and background were both auditory.

Additionally, when looking across subjects, there was a high correlation in response times between the two specific tasks (r = 0.883, p<.05). That is, subjects with longer responses during the visual task also tended to take longer to respond with auditory targets as well (Figure 4, **B**). Note that the relative difference increases consistently across subjects (slope of the regression line is less than 1). Specifically, subjects with the longest auditory RT show the greatest RT decrease (e.g., improvement) in the visual task condition relative to their own auditory performance (Figure 2, increasing shaded region).

**Figure 5:**
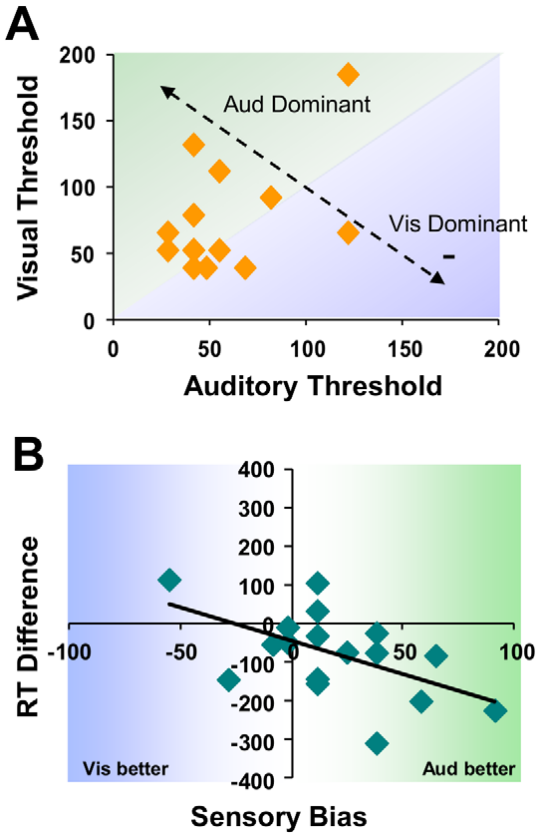
Temporal thresholds and the relation to RT. Temporal thresholds for each modality derived prior to the study (via staircase procedure) show no systematic relationship across subjects (**A**). Shaded areas represent whether a subject shows more temporal acuity in the visual (blue) or auditory (green) domain. There is, however, a high correlation between the amount of sensory bias (difference between visual and auditory thresholds) observed and reaction time differences across subjects (**B**).

Prior to ABR testing, we determined the threshold test stimulus length necessary for each subject to perform the tasks just above chance using an adaptive staircase procedure. The resulting values show that, on average, temporal discrimination thresholds were significantly longer for visual than auditory stimuli (t(16) = 3.23, p<.05), consistent with previous reports [40], [41], and supporting claims that auditory acuity is superior to visual in the temporal domain [42]–[44]. For the group a wide range of temporal thresholds were observed, with no consistent corollary relationship between modalities across subjects (r(16) = 0.079, p>.05, Figure 5, **A**).

It is possible, however, that each subject's *bias* or proclivity towards higher acuity in one modality verses another is directly related to low-level processing. To examine this behaviorally, “sensory bias” was calculated as the difference, in ms, between threshold values for each subject. This is highlighted in the diagonal line in Figure 5, **B**, where high positive values indicate higher acuity to audition than vision (green shaded region, right side), while negative scores denote subjects with a sensory bias towards the visual domain (blue region, left side).

Figure 5 (**B**) shows that the relationship between sensory bias, and the difference in RTs observed between visual and auditory tasks, for each subject. Note that these are highly correlated (r(16) = 0.54, p<.05), such that the degree of sensory bias is predictive of the difference in reaction times between tasks. Specifically, those subjects with a higher temporal acuity in the auditory domain show the largest decrease in response time (a performance benefit) when switching from multiple stimuli occurring within the same modality (auditory task, auditory background) to being across modalities (visual task, auditory background). Likewise, response times by the few subjects with higher visual acuity were increased when the task changed from visual to auditory in nature.

### Relationship between brainstem FFR and performance

Given the observed individual variation in differences in within- modal (*auditory task*) versus cross-modal (*visual-task*) performance and group-level FFR suppression, we sought to establish how task performance was related to the primary metrics of pitch strength and frequency response amplitude, which contribute to ABR signal encoding clarity.

**Figure 6:**
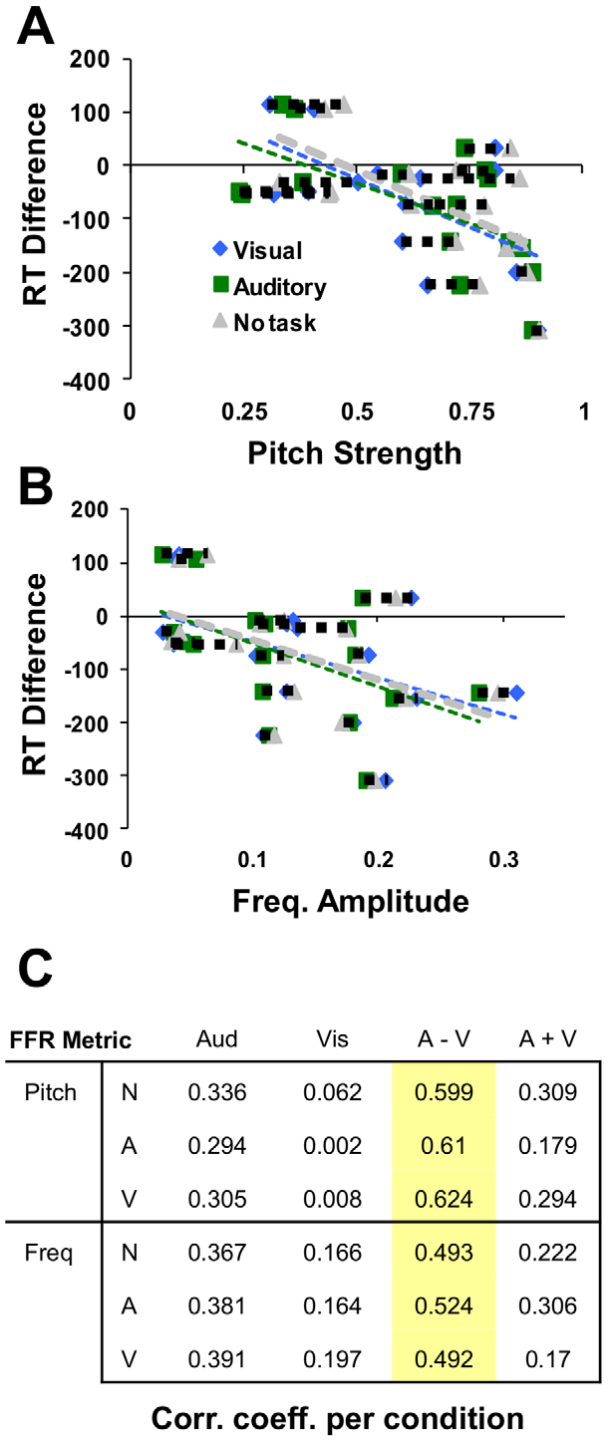
RT changes relative to each FFR metric. Subjects with higher pitch strength (**A**) and higher target frequency response amplitude (**B**) also showed the greatest response time difference (increasingly negative values) when comparing auditory and visual performance. Dashed lines connect each subject's data for the three conditions. This relationship between RT and each FFR metric is consistent regardless of which task (*auditory*, *visual*, or *no-task*, see table in Figure 6, **C**) subjects were performing at the time (similarity of dotted line in Figure 6) Table lists correlation coefficients between response times (columns) and each of the two FFR metrics (rows), as observed within each of the three recording conditions (A = Auditory, V = Visual, N = No task). Highlighted values depict correlations significant at p<.05.

While there was no direct correlation between pitch strength and frequency amplitude with auditory and visual response times by themselves, our primary interest was with the relative difference *between* modalities. Indeed, there was a consistent relationship here – as can be seen in Figure 6, subjects with higher pitch strength (**A**) and higher target frequency response amplitude (**B**) also showed the greatest response time difference when comparing auditory and visual (V–A) performance. Dashed lines connect each subject's data for the three conditions. This relationship between RT difference and each FFR metric is consistent regardless of which task (*auditory*, *visual*, or *no-task*, see table in Figure 6, **C**, which lists correlation coefficients for each pair) subjects were performing at the time (similarity of slopes for dotted line in Figure 6).

**Figure 7:**
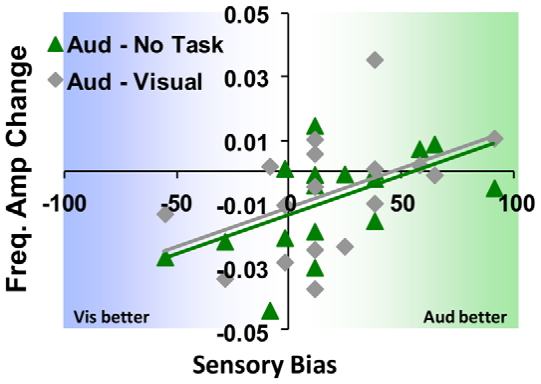
FFR change related to sensory bias. The degree of subjects' sensory bias significantly predicts auditory task-related suppression in primary FFR frequency amplitude (relative to no task), such that those subjects that are more highly acute in the auditory domain show minimal or no suppression. Meanwhile, those whom display less bias or a proclivity more towards vision appear more susceptible to the suppressive effect. A similar trend occurs when comparing auditory against visual tasks, but not for visual above baseline, as well as with FFR pitch strength as the metric.

Above we showed that on average, there was a difference in overall ABR clarity (suppression) with the addition of the task, which is particularly dominant within the auditory modality (observed for both metrics). Meanwhile, participants display a wide range in the degree of sensory bias. Figure 7 shows the relationship between this sensory bias, and the degree of suppression in the amplitude of the FFR primary frequency for the auditory task compared to both *no task* and *visual task* conditions.

Here, the degree of subjects' sensory bias significantly predicts (r(17) = .55, p<.05) suppression in amplitude of the primary FFR frequency from the additional auditory task (relative to no task). A similar trend (r(17) = .46,p<.07) occurs when comparing auditory against visual tasks, but not for visual above baseline (no task, r(17) = .02, p>.05)). That is, subjects with the strongest bias towards vision also showed the largest difference in pitch encoding. A matching, but weaker trend was observed for pitch strength with auditory tasks above baseline (r(17) = .47, P<.06) while not consistently while comparing against visual (r(17) = .34, p>.05) or with visual verses no task (r(17) = .06, p>.05).

## Discussion

Here, we have described a method that allows testing the degree to which background, task irrelevant auditory information is encoded at very early stages of processing, independent of the actual task at hand. With it, we have shown that simply adding a task leads to attenuation of the brainstem frequency following response. Additionally, this low-level modulation is linked to behavioral measures of performance for the primary task and a participant's own perceptual acuity, at least within the temporal domain. Specifically, this study provides three primary results.

First, relative to a baseline of having no task to perform at all, early auditory encoding (brainstem FFR clarity, assessed using two metrics) of background, non-relevant sounds appears inhibited simply by the addition of a sensory-driven primary task. This effect is most evident when the task is also within the auditory domain. While *pitch strength*, a measure of encoding reliability [1], is decreased for visual tasks (a case of operating across modalities) as well, having subjects work within the same modality as the background tone also decreased the spectral amplitude of the brainstem-encoded signal, yielding lower overall encoding acuity. This suggests that the suppression of non-relevant, background information may be stronger when it is within the same modality than when it is across senses.

It is not entirely clear why, although the effects for pitch strength were identical, the frequency amplitude results are not the same for the visual and auditory tasks. One possibility is that this effect is most likely due to differences in the relative sensitivities of each of these methods, and reflect a change occurring within the temporal domain relating to the periodic encoding of the primary frequency (relative to no task). That is, addition of the visual task may induce slight alterations in the temporal encoding of the signal (e.g. jitter) that affect the periodicity of the signal, but are not substantial enough to alter the mean power of the frequency range tested across the entire response window. The auditory task, on the other hand, involves greater perceptual competition and likely induces additional inhibition on early encoding, which decreases the frequency power as well, consistent with visual and auditory processes using different physiological networks for initial encoding.

Second, when looking at subjects' behavioral performance, response times were faster for discriminating visual stimuli than when the same task occurred in the auditory domain, even though the tasks were confirmed to be equally difficult (similar accuracy, set via staircase). While one potential explanation for these differences is that is the neural processes involved in auditory discrimination may simply take more time than visual, which has been espoused previously [38], [39], another possibility is that the facilitation in visual RT over auditory shown here is afforded from operating with stimuli across modalities, rather than within the same sense. Specifically, the auditory condition in this paradigm requires participants to focus on a specific frequency range (e.g. that of the target stimulus) while inhibiting other frequencies (that of the background tones) in order to perform the task, especially since the task target tones and background tones overlap within different, inconsistent periods. In contrast, the visual task likely does not require the same level of focused attention, wherein subjects can simply attend to the visual stream as a whole, and ignore all information within the auditory channel. Much research has suggested that performance is better when dividing attention across senses rather than within (e.g., [45], [46]). This explanation is also supported by the greater overall suppression of the FFR during the auditory than visual task (e.g. Figure 2), suggesting that subjects more actively inhibited the background tone. The difference in response time, which may be viewed as a facilitation yielded from working across, rather than within modalities, was also highly correlated with each subject's difference in temporal acuity, or proclivity towards one sense (sensory bias as measured by differences in threshold discrimination times).

Finally, we have shown that there is a consistent relationship between this cross-modality related facilitation and the measures of low-level auditory acuity utilized here. For both *pitch strength* and *response amplitude* higher values (e.g. better signal consistency or higher SNR) predicted a larger difference in performance time when operating across modalities. That is, subjects who exhibit stronger brainstem encoding also show the greatest improvement in response time when their primary task shifts from operating in the auditory realm to being visual instead. This relationship is fairly robust, occurring regardless of what the subject is actually doing when the FFR is derived.

Perhaps more striking, however, is that the degree to which attenuation in FFR pitch strength (and to a lesser degree frequency amplitude) will be observed when performing an auditory task is directly related to an individual's proclivity towards that same modality. Specifically, those individuals with a higher temporal perceptual acuity within the auditory modality show virtually no change in brainstem encoding quality (when comparing performance on an auditory task to either a visual or no task at all). Meanwhile, participants who are less naturally biased towards the auditory modality also show the largest decrement in signal encoding. This trend does not occur when the task is visual, allowing attention to be focused away from the potentially distracting background tones.

While this study cannot differentiate the neurophysiological source of the suppressive effect described here, it is most likely that this is a top-down (e.g. cortically-driven) phenomenon, and not from the bottom-up. Previous studies have documented cortico-fugal projections which could mediate midbrain (and lower) function [12], [47]–[49]. Additionally, inhibitory effects from the efferent medial olivocochlear (MOC) tracts innervating outer hair cells have been implicated in modulating evoked otoacoustic emissions when attention is focused on other modalities [50]–[52]. This falls in line with the numerous previous reports of auditory [21]–[24] and other cortical [26], [53], [54] modulation from selectively attending to specific stimuli. It should be noted however that it remains controversial whether attention-related mediation of directly driven stimulus-locked brainstem auditory evoked potentials (BAEPs) at the initial stages of encoding should be expected as well [55]–[58] but also see [59].

Mechanistically, we propose that the effects observed here reflect an interplay between brainstem and cortex based on these structural connections. Specifically, the addition of the task increases the total attentional demand within cortex, which leads to a concomitant top-down utilization of the inhibitory cortico-fugal projections, which is turn suppresses the encoding clarity of the FFR. Because the cortical demand is greater in the auditory task (where the background tones are more potentially distracting to the primary task within the same modality), the cortical driver increases the suppression even more, which is reflected is the differential change in frequency amplitude.

The cross-subject variance observed here, which is common in similar studies, certainly represents some combination of differences in individuals' perceptual acuity and whatever strategies they may employ for performing tasks in the presence of background noise. However, accounting for this variance (regardless of its origin) based on participants' perceptual proclivity aides in illustrating differences in sub-populations which might otherwise not be known.

These results provide important implications within two avenues. First, we have established the utility of a paradigm which allows the ability to compare brainstem-level effects relative to different types of cortical tasks, while remaining independent of the stimuli used for those tasks. Unlike previous approaches using the response-eliciting stimuli as a primary component of the task, ABR time-locked events in this case are completely non-task relevant and temporally separate from task-related events. In our analyses we included only data from time periods non-overlapping with the primary task stimuli or response periods, so that the EEG-measured activity examined here reflects only time *between* task trials and the response to the background tone, and thus all stimulus features were physically identical across all conditions. This means that any effects observed must be related to chronic, global state-mediated changes rather than stimulus-driven, bottom-up or immediate response influences.

It should be noted that in this paradigm, we cannot completely separate whether the fluctuations observed here stem solely from the brainstem FFR, or alternatively from a concomitant increase in cortical or other sources related to performing a task which could change the overall background electrical field level assumed to be a baseline. That is, although the target signal discussed here is the brainstem FFR, it is possible that the suppressive effect observed with our metrics appear as a result of increased “noise” (meaning non-brainstem originating fluctuations) adding variance to the overall signal during the active task conditions. For instance, cortical oscillations are known to occur for sustained periods of task engagement [60], [61], and although oscillations differ across sensory modalities, they are critical for binding across senses [62].

However, while it is indeed likely that parallel cortical activity is induced by the task and maintained between trial periods, we do not feel this could fully explain the extent of the effects examined here. The highpass filter (80 Hz) should remove typical cortical activity, although this will not eliminate all neurophysiologically driven signals such as microsaccades. Perhaps more importantly, because the techniques here rely on averaging across hundreds of stimulus epochs, any remaining contaminating effect would require the oscillations to be phase-locked with the stimuli in order to survive combining across so many trials. Additionally, any such sources would have to also fall within the specific frequency range targeted in our analyses (220 Hz). Although we did observe small differences in the pre-stimulus period, these were not statistically significant at the group level or correlated with any of the behavioral measures compared here, such as the observed relationship between each participant's sensory proclivity and the extent of suppression observed in the FFR (Figure 7).

Second, we have shown that variance in the quality of sound encoding sub-cortically can be introduced simply by the addition of a task. Not only is the degree to which this occurs dependent on the type of task and modality used, but also appears to vary across individuals based on other intrinsic properties, such as their own discrimination acuity within a particular sense. This suggests that care should be taken in future brainstem FFR-based studies, because the size of the effect observed may depend more on external parameters of the study than originally assumed. Likewise, excess variance due to individual differences in susceptibility to these effects could decrease the overall power of a study to properly identify hypothesized effects.
